# Lysozyme and DNase I loaded poly (D, L lactide-co-caprolactone) nanocapsules as an oral delivery system

**DOI:** 10.1038/s41598-018-31303-x

**Published:** 2018-09-03

**Authors:** Omar S. Abu Abed, Cheng Chaw, Lee Williams, Amal A. Elkordy

**Affiliations:** Department of Pharmacy Health & Well-being, Faculty of Health Sciences and Wellbeing, University of Sunderland, Sunderland, SR1 3SD UK

## Abstract

Clinical applications of oral protein therapy for the treatment of various chronic diseases are limited due to the harsh conditions encounter the proteins during their journey in the Gastrointestinal Tract. Although nanotechnology forms a platform for the development of oral protein formulations, obtaining physiochemically stable formulations able to deliver active proteins is still challenging because of harsh preparation conditions. This study proposes the use of poly (D, L-lactic-co-caprolactone)-based polymeric nanocapsules at different monomers’ ratios for protein loading and oral delivery. All formulations had a spherical shape and nano-scale size, and lysozyme encapsulation efficiency reached 80% and significantly affected by monomers’ ratio. Trehalose and physical state of lysozyme had a significant effect on its biological activity (P < 0.05). Less than 10% of the protein was released in simulated gastric fluid, and 73% was the highest recorded accumulative release percentage in simulated intestinal fluid (SIF) over 24 h. The higher caprolactone content, the higher encapsulation efficiency (EE) and the lower SIF release recorded. Therefore, the formulation factors were optimised and the obtained system was PEGylated wisely to attain EE 80%, 81% SIF release within 24 h, and 98% lysozyme biological activity. The optimum formulation was prepared to deliver DNase, and similar attributes were obtained.

## Introduction

Drug delivery through the oral route of administration is the most common and acceptable systems due to their non-invasive and cost-effective properties. However, it is challenging to deliver the therapeutic proteins orally due to different hurdles; extreme stomach acidity, enzymatic degradation, physiological (permeability) barriers, and physicochemical instabilities of proteins^1,2^. Although several novel protein delivery approaches have been developed, the parenteral dosage form is still the most common one^2^. Nanotechnology-based drug delivery systems have the potential to deliver therapeutic proteins orally^3^. The development of nanocarriers, e.g. liposomes, noisomes, and polymeric nanoparticles was amongst the promising approaches attempted to overcome the obstacles facing oral protein delivery^4–6^. Moreover, several studies revealed that nanoparticles have an enormous impact on the oral bioavailability of biologic drugs including therapeutic proteins^3,4,7–9^. Polymeric nanocapsules (PNCs) comprise a core-shell structure in which proteins are confined and surrounded by a biodegradable polymer^10,11^. Nanocapsules system requires less amount of polymer contents to protect the vulnerable drugs^12^ and has the higher efficiency to encapsulate the drugs due to enhancing drugs solubility in the nanocapsules cavity^13^. Moreover, the revolution of polymer synthesis technology led to the development of aliphatic polyesters polymers which are biocompatible and biodegradable, therefore, less accumulation in tissues and less immune rejection^14^.

Polymeric nanocapsules are usually used in protein therapy to deliver the therapeutic proteins and improve their pharmacokinetics properties^15^, hence, reducing the frequency of administration and protecting them from GIT harsh conditions. Various material and process parameters may affect the attributes of nanocapsules, e.g. rate of drug release or the percentage of encapsulation efficiency. Therefore, selecting proper polymers is a crucial step in formulating the nanocapsules; due to their role in the determination of release rate, the biological activity of the encapsulated proteins, and the encapsulation efficiency^16,17^. Polylactide (PLA), polycaprolactone (PCL), polyethene glycol (PEG), polyglycolic acid (PGA), their copolymers are the most commonly used biodegradable and biocompatible polymers for drug delivery purposes as they showed the promising results in term of biodistribution^16,18,19^. Despite the high efficiency of PCL and its copolymers to confine the drugs inside them, their release profiles of macromolecules are poor, mainly when the copolymer comprises of PCL and another hydrophobic polyester polymer, e.g. PLA. The low percentage of protein release from PCL NCs is attributed to their innate hydrophobicity and permeability for small molecules but, not for macromolecules, because of the tiny pores on the polymers’ surface^20^. Encapsulating therapeutic proteins in polymeric nanocapsules is accompanied by several challenges. Denaturation of encapsulating protein is considered the most common drawback of protein loaded polymeric nanocapsules^21^. This denaturation may be caused by the harsh formulation process parameters and materials, e.g. sonication or the used organic materials^22,23^. Insertion of extremolyte sugars showed stabilising effects for the encapsulated proteins against the destabilising conditions; e.g. the biological activity of asparaginase was improved by adding trehalose to the inner protein solution^24^. However, neither trehalose nor sucrose has any stabilising effect on lysozyme conformational and biological stability^25,26^. The failure of extremolytes to protect proteins may be attributed to several factors; their concentrations, the nature of polymers, or the ability of the polymers to confine the protein which may allow the leakage during and after the formulation process. Therefore, obtaining high encapsulated protein stability along with high encapsulation efficiency, high SIF release within the desired time, and protection against the gastric conditions is challenging. Thus, optimising these individual factors is crucial to attaining successful oral formulations.

To our knowledge, no studies so far have investigated the amount and physical state of trehalose required to prevent the denaturation of encapsulated proteins. Moreover, this is the first report utilises poly (D, L lactic-co-caprolactone) PLC to prepare PNCs intended for oral delivery. A copolymer comprised of lactide and ε-caprolactone was selected for their safety, biocompatibility biodegradability and enhanced pharmacokinetic properties as stated by^27^.

In this work, lysozyme/DNase-loaded polymeric nanocapsule formulations were prepared by modified double emulsion/evaporation method. The formulation plan of this study was according to a full design of experiment where the effect of different ratios of PLC monomers, and the quantity of added trehalose on solid and liquid lysozyme on the physicochemical properties of NCs, lysozyme release in SIG and SGF, and the biological activity of lysozyme was investigated. All factors were optimised, and PEGylated optimised formulation containing DNase as a therapeutic protein was prepared and characterised.

## Results and Discussion

### Formulation and physicochemical characterisation of polymeric nanocapsules

**Figure 1:**
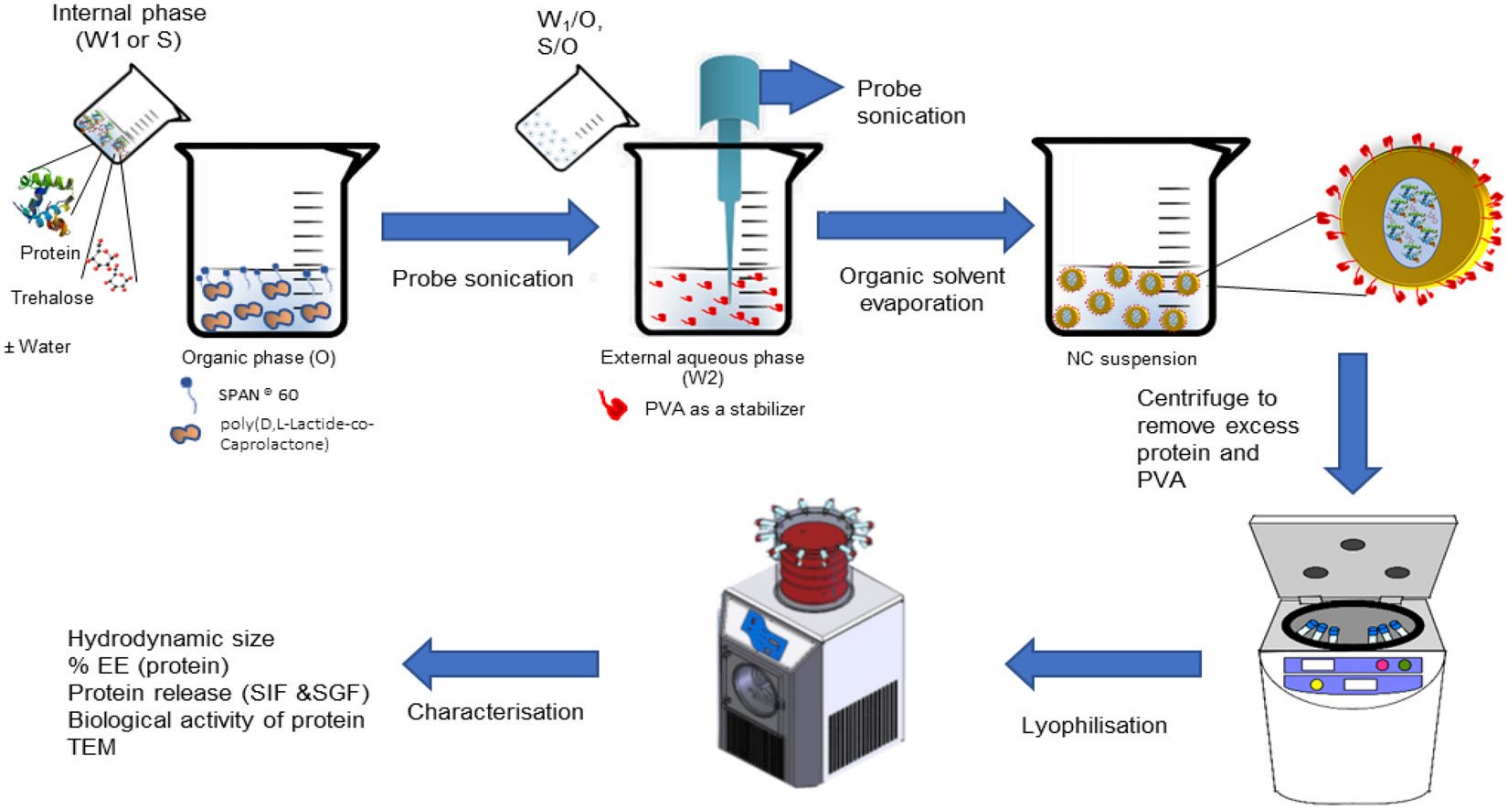
Preparation of PNCs by double emulsification (w/o/w or s/o/w)/solvent evaporation method. Schematic representation of the formulation method of the protein-loaded PNCs. The PNCs were formulated by the emulsification of an internal aqueous phase (Protein and trehalose in water)/suspending of internal solid protein and trehalose into organic phase (ethyl acetate) containing polymers, and Span® 60. Then, further emulsification step has taken place between the internal (w/o or s/o) and an external aqueous phase (water, and PVA) using probe sonication. The solvent is removed forming PNCs aqueous dispersion. Finally, the formed suspension was lyophilised over 48 hours.

Lysozyme containing PNCs were formulated using poly (D, L-Lactide-co-Caprolactone) (PLC) at two different ratios (86:14 or 40:60) to encapsulate protein in liquid or solid state with different trehalose concentrations (1 mM and 10 mM). All factors were investigated together in a full factorial design of experiment rather than testing one factor at a time. All formulations were prepared either by S/O/W or W/O/W methods, Fig. 1.

**Figure 2:**
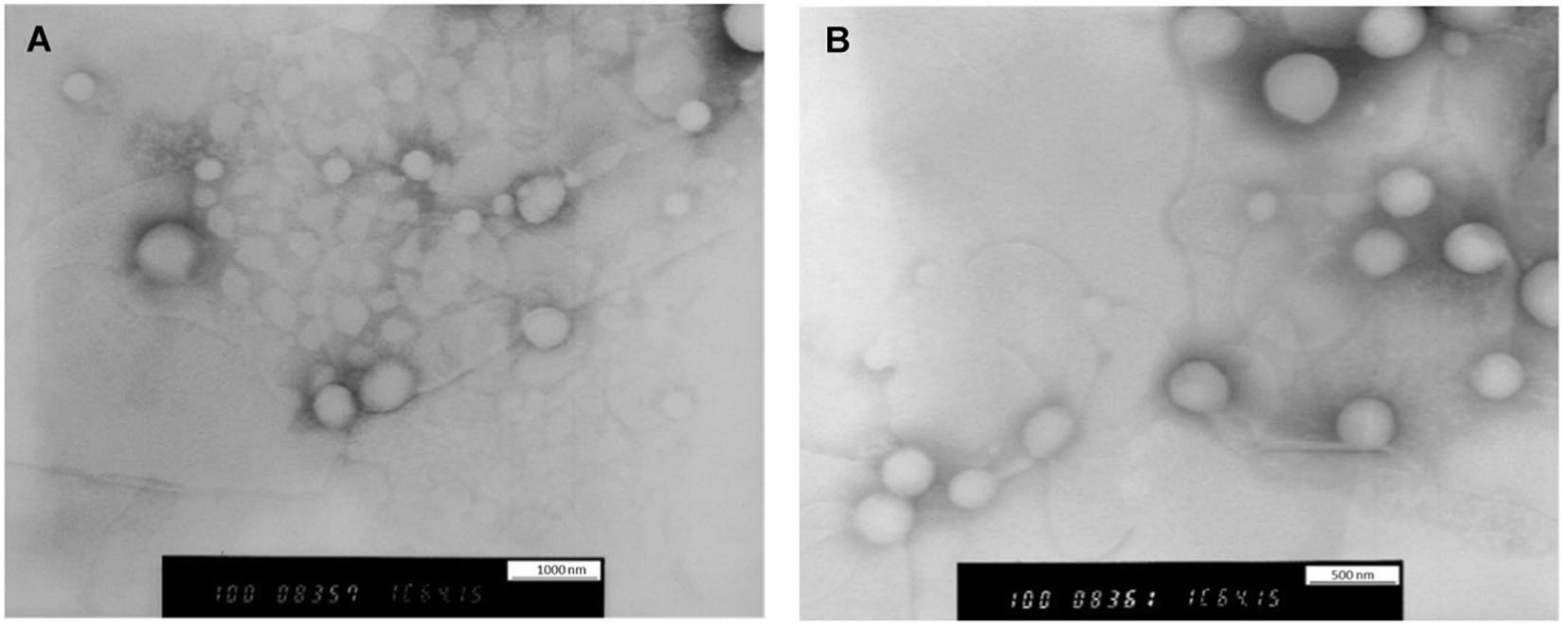
Morphological examination polymeric PNCs by utilizing TEM. PNCs were prepared by double emulsification/solvent evaporation method. The images were taken by using negative staining technique (sodium silicotungstate solution). (**A**) TEM image for NC7 prepared by encapsulating solid lysozyme and 10 mM trehalose in (86:14) PLC. (**B**) TEM image for NC3 prepared by encapsulating solid lysozyme with 1 mM trehalose in (86:14) PLC.

Investigation of nanocapsules morphology by TEM revealed round core-shell structure with smooth external surfaces and particles of various size. Some of the agglomerates were also shown in the images with no noticeable difference in the shell thickness was noticed. The presence of the spots at the core of PNCs is supportive of the hypothesis that trehalose is encapsulated inside the polymeric shell. The spotty core was absent in the formulations prepared with 1 mM trehalose, Fig. 2. Also, trehalose encapsulation was confirmed by utilising HPLC to detect peaks of trehalose. Data are provided in the supplementary information.

The hydrodynamic size obtained by dynamic light scattering was in the wide range of (325.3–865.3) nm, particle size distribution curves are provided in the supplementary information. None of the copolymers blocks ratio or the preparation method led to a statistically significant difference in PNC particle size (P > 0.05). However, high trehalose concentration increased PNC diameters (601–865 nm) significantly (P < 0.049), Table 1. This could be due to the accumulation of both protein and trehalose together in the core which may acquire a more substantial inner space. This assumption is supported by the observed spotty core in the presence of trehalose in the formulations, Fig. 2.

**Table 1:** Optimisation of the lysozyme-loaded NC formulations (model protein) and their physicochemical properties.

ID	Trehalose (mg)	Core physical state	Caprolactone/D, L Lactide ratio	Size (nm)^a,b^	PDI^a^	%EE^c,b^	Drug release (SGF)^d,b^	Drug release (SIF)^e,b^	BA^f^	LE%^g^
NC1	1	Liquid	14:86	467.1 ± 11.8	0.383	32.1% ± 2.8	13.05% ± 0.8	72.53% ± 4.0	43.56	1.28% ± 0.11%
NC2	1	Liquid	60:40	517.9 ± 46.7	0.384	68.4% ± 1.5	10.73% ± 4.0	32.38% ± 2.8	39.70	2.74% ± 0.06%
NC3	1	Solid	14:86	378.4 ± 32.5	0.335	39.3% ± 1.5	7.92% ± 1.9	64.36% ± 0.8	56.53	1.57% ± 0.06%
NC4	1	Solid	60:40	325.3 ± 14.5	0.291	62.3% ± 2.6	11.05% ± 1.9	30.95% ± 1.8	58.96	2.49% ± 010%
NC5	10	Liquid	14:86	831.9 ± 29.6	0.388	31.3% ± 2.7	10.84% ± 3.2	64.90% ± 2.4	79.24	1.25% ± 0.11%
NC6	10	Liquid	60:40	865.3 ± 22.6	0.382	69.2% ± 5.3	10.84% ± 2.9	38.52% ± 3.7	77.24	2.77% ± 0.21%
NC7	10	Solid	14:86	601.1 ± 9.7	0.346	41.5% ± 1.9	10.10% ± 3.4	68.53% ± 0.9	95.63	1.66% ± 0.08%
NC8	10	Solid	60:40	627.2 ± 39.6	0.366	64.1% ± 1.3	10.57% ± 3.5	35.68% ± 4.4	97.42	2.56% ± 0.05%

^a^Size and PDI were measured by dynamic light scattering in deionised water.

^b^Results are expressed as mean ± SD (n = 3).

^c^% Encapsulation efficiency (%EE) of protein was determined by measuring absorbance at ƛ 21 nm in a size exclusion liquid chromatography. %EE was determined using the following equation:

%EE = (amount of encapsulated protein/initially used protein) ∗ 100%.

^d^% of total protein release in SGF after 4 hours.

^e^% of total protein release in SIF after 4 hours.

^f^Biological activity was assessed by measuring the ability of encapsulated lysozyme to lysis the bacterial cell wall.

^g^% Loading efficiency (%LE) of nanocapsules was calculated by using the following equation:

%LE = (amount of encapsulated protein/amount of polymer used) * 100%.

Unencapsulated protein was removed by centrifugation and, then, freeze-dried for 48 hours before finding the percentage of encapsulation efficiency. Protein encapsulation efficiency (EE%) for all formulations was quantified by size exclusion chromatography and described in Table 1. Values of EE% were obtained over a wide range (31.3–69.3%) and significantly affected by the ratio of monomers (P < 0.0106). The higher ratio of ε-caprolactone the higher noticed EE%. Although trehalose and the physical state of proteins had no significant effect on EE%, utilised DOE revealed a significant effect caused by the interaction between the physical state and copolymer ratio. Thus, a substantial increase in encapsulation efficiency was recorded when (40:60) PLC was used to encapsulate liquid lysozyme when compared to its ability to encapsulate lysozyme in the solid state. High EE% caused by the presence of a high ratio of ε-Caprolactone refers to the longer hydrocarbon chain of ε-Caprolactone, which helps to confine the protein and reduce the leakage to the outer aqueous medium during the emulsification process. Longer hydrocarbon chain accelerated the solidification of nanocapsule shell during organic phase evaporation. This rapid solidification could increase the polymer ability to confine the protein and reduce the amount of the leaked protein into the external aqueous medium.

### *In vitro* release of lysozyme in Simulated Gastric Fluid (SGF) and Simulated Intestinal Fluid (SIF)

**Figure 3:**
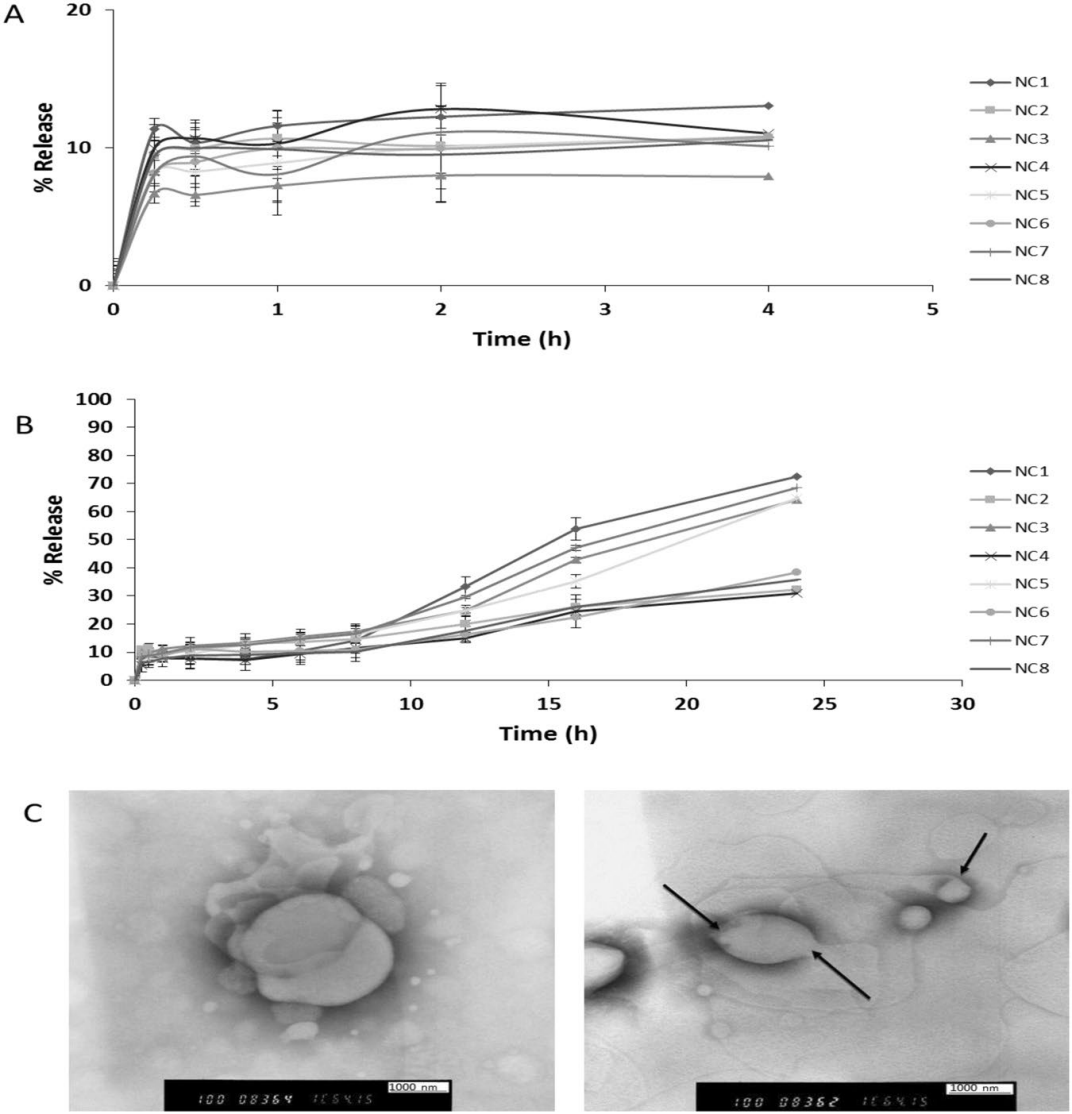
*In vitro* release profile of lysozyme from PNCs in simulated gastrointestinal fluids (without enzyme) at 37 °C. Lysozyme release from PNCs was determined in simulation gastric fluids (SIF) and simulation intestinal fluid (SGF) for 4 and 24 hours, respectively. Both SIF and SGF were prepared according to British Pharmacopeia. However, no digestive enzymes were added. Protein release was determined by quantification of protein amount remaining in pellets by using SEC after pellets disruption and subtracting the remaining from the original value (100 μg) to find out release percentage. (**A)** The release profile of lysozyme in SGF over 4 h, where diphasic release pattern was noticed. (**B)** The release profile of lysozyme in SIF over 24 h, where triphasic release pattern of burst, plateau, and sustained release was noticed. (**C**) TEM images of PNCs after protein release. The images reveal the mechanism of drug release and ensure that no polymer erosion has happened.

The release of lysozyme from the polymeric Nanocapsules was investigated over 4 and 24 hours in SGF and SIF, respectively (Fig. 3), and the total percentage of lysozyme released is shown in Table 1. In SGF, release process was biphasic over 4 hours when an initial release phase (8%) was followed by an equilibrium state or slower release with an average overall release around 10.5% for all formulations (Fig. 3A). Whilst, a triphasic pattern represented the release of protein from the PNCs’ reservoir into SIF over 24 hours, which is concluded by; the initial burst phase of the proteins (8%) within the first 15 minutes. Then, a plateau for 8–10 hours reflecting equilibrium or a slow diffusion state in the second phase. Finally, sustained released was noticed over the rest of 24 hours (Fig. 3B). The secondary structure of released lysozyme in acidic and alkaline media was investigated by FTIR; the results revealed that lysozyme integrity was not affected after the release. FTIR data are included in SI.

Lysozyme release occurred via different mechanisms; the initial burst phase may have happened due to the adsorbed proteins at the PNCs surface. The second release phase or the plateau phase has resulted when adsorbed lysozyme on the inner polymer surface had diffused out^28^. Afterwards, the last sustained release phase usually results due to various mechanisms, e.g. particle structures weakening due to plasticising effect, or polymer degradation and subsequent matrix erosion. Also, diffusion out of the polymer due to various causes, e.g. formation of some pores or water channels within polymer shells as medium penetrated the matrix particles may be the drug release mechanism.

In this study, the shape of nanocapsules was inspected just after the release by using TEM to figure out any shape changing in the nanocapsules structure. The spherical shape of PNCs was retained after protein release; however, the particle size was increased with some distortion of the particle surface, Fig. 3C. TEM images confirm that; no erosion occurred in the copolymers, and the release happened due to diffusion of the protein outside through the polymeric shells. When the PNCs were immersed in the aqueous medium (SIF), as direct contact between the polymeric outer shell and the aqueous medium plasticizes the polymer chains^29,30^, This allows further fluid influx into the inner core of the PNCs and eventually dissolved the protein and helped the PNCs to swell and generate some pores and increased the permeability due to its weakened and plasticised mechanical properties. Consequently, lysozyme started to efflux outside in a sustained manner with time.

The explanation was consistent with the study conducted by *Blasi et al*.^29^. *Blasi et al*.^29^ stated that; T_g_ of PLGA had been dropped by incubating in water at two different temperatures, i.e., 23 °C and 30 °C. Moreover, the same study showed that the decline in T_g_ was 15 °C after 1 hour of incubation irrespective of temperatures used. This depletion in T_g_ value has resulted in converting the polymer structure from the glassy state into a rubbery state, thus, increased the polymer fluidity, which consequently, may increase the drug release from polymeric drug delivery systems.

Statistical analysis revealed that total lysozyme released in SIF was significantly affected by the monomers’ ratio, where (86:14) PLC attained higher drug release over 24 hours.

D, L-lactide is the racemic form of Lactide moiety due to the presence of chiral methyl group in the Lactide. The molecule backing of racemic Lactide forms changed the mechanical properties from being crystalline or semi-crystalline to utterly amorphous form for the copolymer, which subsequently, decreases the polymer consistency. This changing in internal structure decreases T_g_, thus plasticizes the polymer and increases the permeability. The Poly (L-Lactide) form has 35:65 of crystalline: amorphous, while poly (D, L-Lactide) is entirely amorphous has a T_g_ value of 57 °C in comparison to pure poly(L-Lactide) (65 °C); which reflects different mechanical properties. Furthermore, forming copolymer can create new molecules with different mechanical properties, which may be able to reduce the rigidity of the polymers as described by^31^.

PLC 86%:14% has low T_g_ (16 °C)^32^. This depletion in T_g_ means that the copolymer gets plasticized and become rubbery at temperatures higher than 16 °C (body or release experiment temperature is 37 °C). However, 40:60 PLC has a melting temperature (Tm) of 31 °C^32^. Hence, mentioning melting temperature rather than glass transition temperature reflects that the polymer mainly consists of dominantly crystalline or semi-crystalline structure^33^. This means the polymer has more rigid and stable bond backing, thus confining lysozyme inside the polymeric system for a longer time due to slow release. The slow drug release of (40:60) PLC is caused by its slower softening rate when exposed to the dissolution condition at 37 °C when compared to (86:14) PLC.

### The biological activity of encapsulated lysozyme

**Figure 4:**
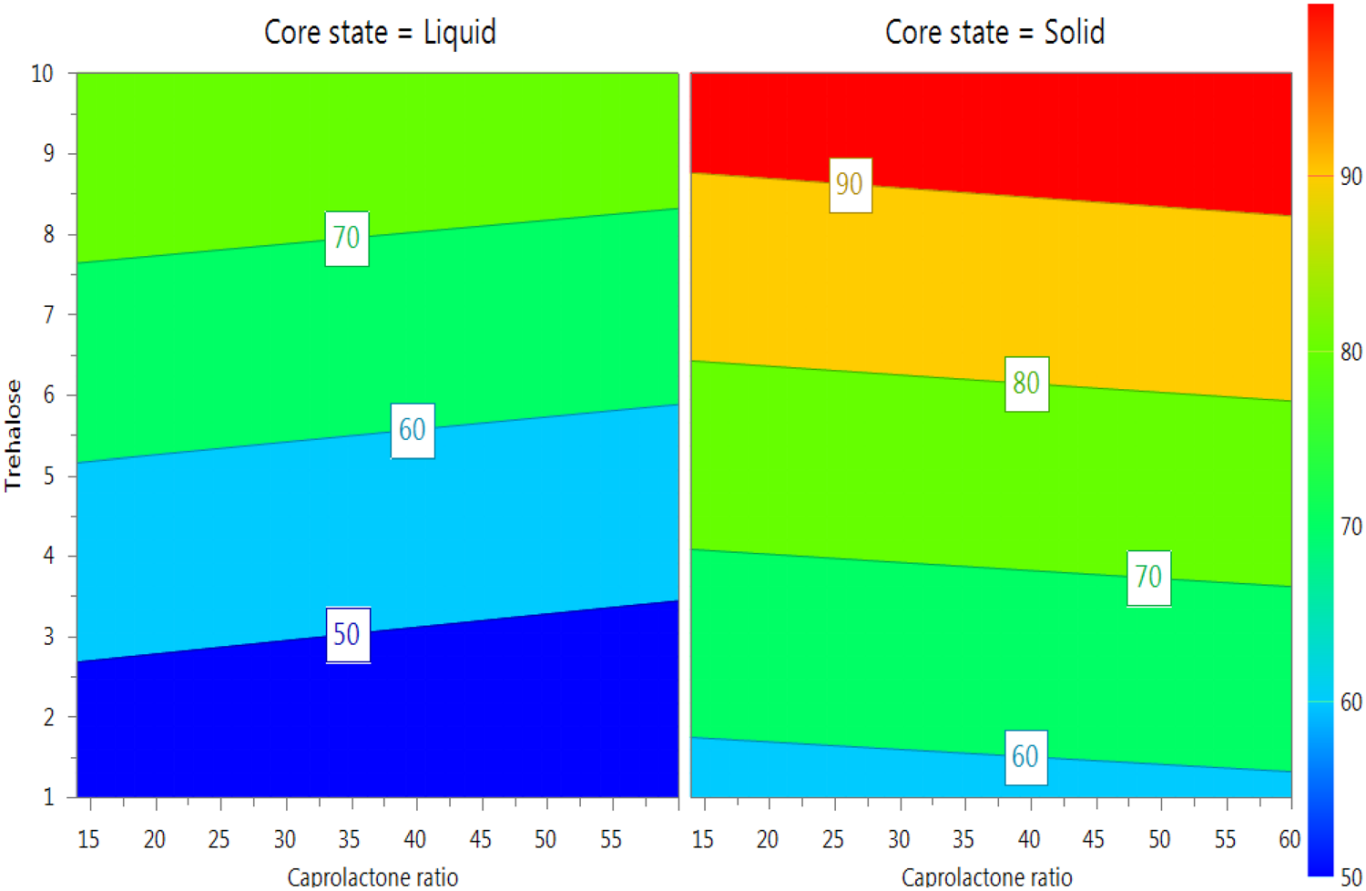
4D Response contour plot illustrating the effect of the investigated factors on the biological activity of lysozyme. The biological activity of lysozyme was measured by applying a predefined enzymatic assay which determines the ability of lysozyme to lysis the bacterial cell wall by breaking the b-1, the 4-glycosidic linkage between N-acetylglucosamine (NAG) and N-acetyl muramic acid (NAM) in *Micrococcus lysodeikticus*. UV-visible spectroscopy was utilised to record the decrease in A450 over 5 minutes. The response plot illustrates the difference in biological activity between solid and liquid encapsulated lysozyme. Also, it shows how increasing the amount of used trehalose protected lysozyme significantly. Blue colour represented the least protected lysozyme (least biological activity), while, the red scale is for lysozyme retained high biological activity after encapsulation.

Encapsulated lysozyme retained 39.69–97.42% of their original activities, Table 1. Biological activity was increased significantly with increasing the amount of trehalose (p-value < 0.011) and encapsulating lysozyme in the solid state (p-value < 0.003), Fig. 4. Stability of lysozyme in solid state refers to the low rate of hydrolysis due to the absence of water^34^. Presence of water around lysozyme can cleave amide bonds and thus, denature the protein during the process of preparation^35^. The rate of chemical reaction of protein in the solid state is minimum due to the internal zero energy which protects the physical and chemical integrity of proteins^36^. Trehalose stabilises the proteins in solutions by preferential hydration mechanism and reducing the protein mobility which keeps the protein in the folded state^37,38^. Moreover, trehalose stabilises proteins by working as a water substitute during the lyophilization process by maintaining proteins’ hydrogen bonds, which eventually reduces the unfolding and protein deactivation^39^.

Trehalose stabilises solid proteins by so-called “Glass Dynamic Hypothesis”. The glass dynamic hypothesis states that trehalose forms rigid and inert solid filler around the proteins, which separates the protein molecules and inhibits any chance of protein motion and collision with other protein molecules. This eventually restricts the proteins unfolding and denaturation^40^.

### Optimisation of formulation factors to prepare lysozyme/DNase-PNCs with optimum characteristics

**Figure 5:**
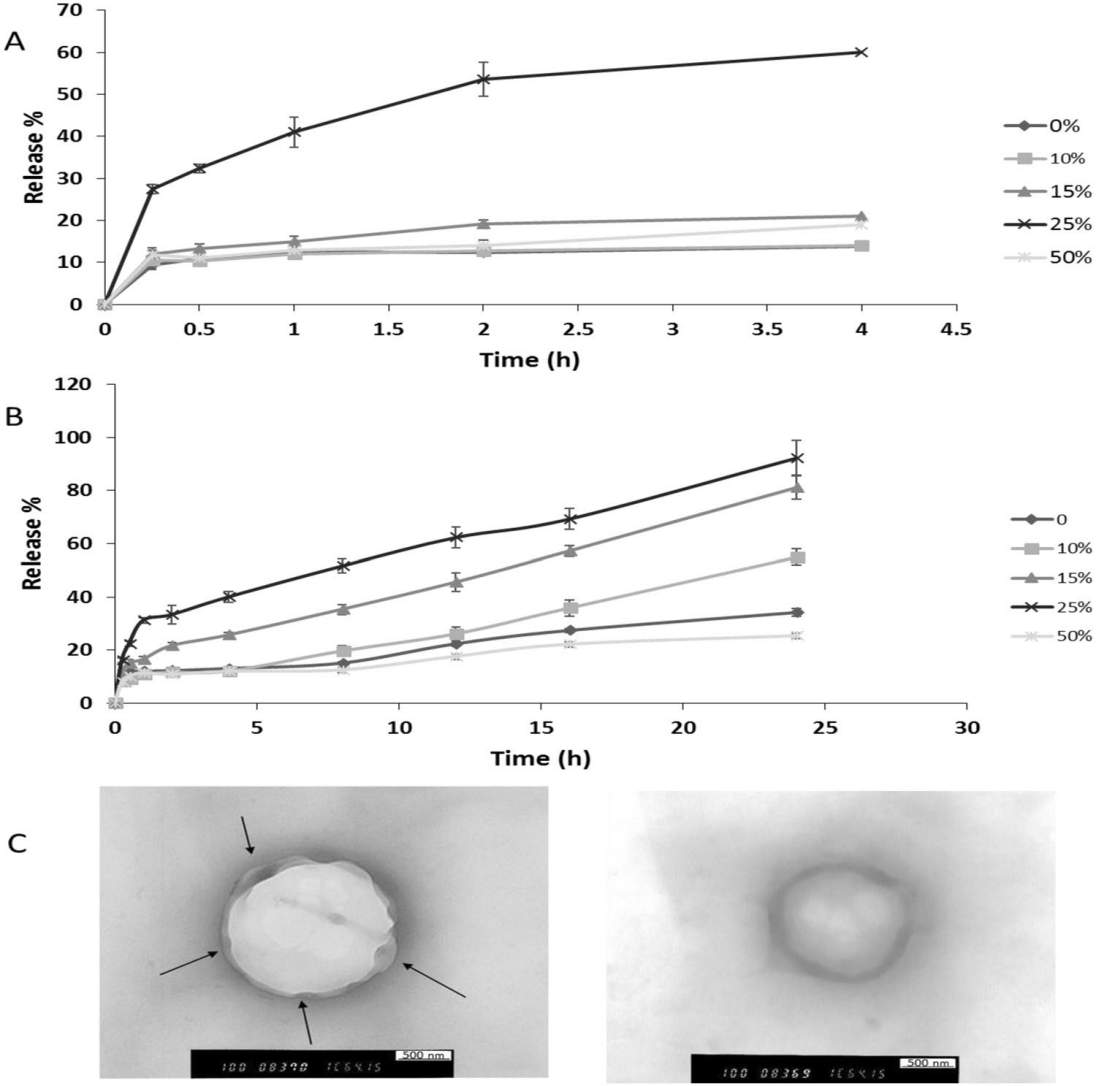
*In vitro* release profile of lysozyme from PEG-PNCs in simulated gastrointestinal fluids (without enzyme) at 37 °C. Lysozyme release from PEG-PNCs was determined in simulation gastric fluids (SIF) and simulation intestinal fluid (SGF) for 4 and 24 hours, respectively. Both SIF and SGF were prepared according to British Pharmacopeia. However, no digestive enzymes were added. Protein release was determined by quantification of protein amount remaining in pellets by using SEC after pellets disruption and subtracting the remaining from the original value (100 μg) to find out release percentage. PEGylation was by adding a different amount of PEG 8000 to the prepared formulations but before freeze drying. Percentage of PEG means the percentage of the weight of added PEG relatively to the weight of the initially used polymer. (**A)** The release profile of lysozyme in SGF over 4 h, where 25% PEG was adding has increased the release in SGF to around 60%. (**B)** The release profile of lysozyme in SIF over 24 h, when using of 15% and 25% of PEG resulted in very high drug release. (**C**) TEM images of 25% PEG-PNCs after protein release in SIF. The image demonstrates the large pores formed at the surface and facilitated the release of lysozyme. (**D**) TEM images of 50% PEG-PNCs after protein release in SIF. The image illustrates the formed shell around PNCs which is expected to have hindered lysozyme release.

Altogether no formulation appeared to have optimum properties. Therefore, an optimising equation describing the best-fitted model was applied to optimise the factors to find the best-compromised responses. The optimised formulation is predicted to be prepared from 40:60 poly(D, L-Lactide-co-Caprolactone) to encapsulate solid lysozyme with 9 mM trehalose to obtain the following predicted responses; 70% EE% and 95% biological activity. However, release profile was predicted to stay low (36.5%) over 24 hrs even after optimisation; this is expected as high content PCL-NCs showed slow release kinetics. Several approaches could increase the release; however, not all of them are suitable for the other physicochemical properties. PEGylation is one of the most commonly used approaches in the preparation of polymeric nanocapsules due to its biocompatible nature and its ability to enhance drug release^41,42^.However, PEGylating time is critical as blending the used polymers with a hydrophilic polymer, e.g. PEG from primary stages may significantly reduce the encapsulation efficiency due to the leakage of protein into the outer aqueous phase during the process of the formulation^43^. Therefore, PEG 8000 was selected due to its relatively high molecular weight and was added after the preparation and solidification of PNCs and just before the lyophilisation. The quantity of added PEG was optimised by adding different amounts to the formulation, Fig. 4. Four different formulations were prepared, and PEG was added in different quantities; 10%, 15%, 25%, and 50%. The highest drug release in SIF was 92% and observed for PEG-PNC_25%_ containing PEG, while 60.02% of lysozyme was released from the same formulation in SGF. Nevertheless, the release of SIF from PEG-PNC_15%_ was 81.06%, but it suits the oral delivery more, as the release in SGF was 21.06%. Lysozyme release from the PEGylated and control (non-PEGylated) formulations was in the diphasic pattern except the release from PEG-PNC_25%_ in SIF where triphasic was observed, Fig. 5.

Enhancing the systems’ release was caused by PNCs interaction with PEG which softened the polymer by increasing the water content it. Caprolactone is permeable to small molecules but, not macromolecules like protein because of the tiny pores on its surface^20^. PEG8000 may have increased the already existing pores by improving the permeability. However, increasing the quantity of PEG8000 to 50% w/w decreased the release percentage, and even less than the control formulations. After finding out the PEGylated PNCs morphology by TEM, a shield of PEG was formed around the particles which may hinder the release significantly, Fig. 4C.

Two PNCs optimised PEGylated formulations (15% PEG8000) were prepared and characterised for encapsulation efficiency, drug release and biological activity. The obtained results were close to the predicted ones, where %EE 74.6%, 97% biological activity, and 82.15% SIF release were obtained.

Lysozyme was used as a model protein throughout the study. After optimising the factors and PEGylating the optimum formulation, PEG-PNCs_15%_ was prepared to deliver deoxyribonuclease I (DNase I) and then characterised. DNase is a therapeutic protein being used for the treatment of cystic fibrosis. The biological activity of encapsulated DNase I exceeded 95%, which reflects the ability of DNase to withstand during the process procedures. Interestingly, the %EE, release profile in SIF, and release profile in SGF are comparable to those obtained from encapsulating lysozyme into PNCs at the same optimum conditions. The developed PNCs may present an excellent model for developing future polymeric nanocapsules containing other macromolecules, e.g. therapeutic proteins, monoclonal antibodies, and genes.

## Conclusion

This is the first report on the investigation of trehalose quantity role in protecting proteins during the polymeric nanoencapsulation processes. Moreover, poly (D, L lactide-co-caprolactone) was utilised to prepare the PNCs for oral delivery, which also has never been studied before for oral delivery. Design of experiment was built to examine the effect of material attributes and optimise them to obtain PNCs with the desired %EE, release kinetics, particle size, and biological activity of proteins. The optimised formulation was capable of (i) retaining the high biological activity of encapsulated proteins, (ii) releasing high percentage of active proteins in SIF, (iii) obtaining a high percentage of encapsulation efficiency, and (iv) protecting the protein from degradation in gastric fluids. Herein, this data suggests that the biological activity of the protein was increased from 39% to 97% when the amount of trehalose used was increased from 1 mM to 10 mM. Also, high PCL content-copolymer could confine high percentage of protein. However, the extended release was observed. Therefore, high PCL-content PNCs was used to prepare the optimised formulation with using PEG as a release enhancer. PEGylated PLC-NCs confined more than 80% of the used protein and released 80% of the encapsulated protein in SIF. PEGylated PLC-NCs could be a promising nanocarrier system for oral delivery of therapeutic proteins. Lysozyme was used as a model protein throughout the study, in addition, PNCs containing DNase, as a therapeutic protein, were prepared at the optimised conditions.

## Experimental Sections

### Materials

Lysozyme (Mucopeptide N-acetylmuramyl hydrolase, Muramidase, lyophilized powder, ≥40,000 units/mg protein) obtained from chicken egg white, was provided by Sigma-Aldrich. Deoxy Ribonuclease I (DNase I lyophilized powder, ≥400 Kunitz units/mg protein) obtained from bovine pancreas, was provided by Sigma-Aldrich. Ethyl acetate, polyvinyl alcohol PVA, Span^60®^, DNA, Micrococcus lysodeikticus, pepsin, Poly (D, L-Lactide-co-Caprolactone) (40:60), Poly (D, L-Lactide-co-Caprolactone) (86:14), polyethylene glycol 8000 (PEG 8000), and hydrochloric acid were purchased from Sigma–Aldrich Co. (Poole, Dorset, UK). Trehalose dihydrate was purchased from VWR Co., (Radnor, PA, USA). Electrochemical analysis grade acetonitrile was purchased from Fischer Scientific, (Loughborough, UK). Nano pure water (>Ω 18, Milli-Q) was obtained from a Diamond Lab Water System (Triple Red Laboratory Technology, Bucks, UK).

### Design of Experiment

The design of experiment was selected to connect the overall quantitative data in the experiment in a lucid way. Therefore, the full experimental design was generated combining three factors each one has two levels to form eight different possibilities (2^3^). The effect of the factors on the desired criteria (responses) was assessed by the preparation of formulations in triplicate and then characterise them in the light of the suggested qualities to screen the significance of each factor thus optimise them by utilising multilinear regression (MLR), Equation 1.

1 $${\rm{y}}={\rm{\beta }}0+{\rm{\beta }}1\times 1+{\rm{\beta }}2\times 2+{\rm{\beta }}12\times 1\times 2+\ldots +{\rm{\varepsilon }}$$

MODDE 10.1 software (Umetrics AB, Umea, Sweden) was utilised for statistical analysis.

### PNCs preparation

Polymeric nanocapsule formulations were prepared by the double emulsion solvent evaporation method via preparing water in oil in water (W/O/W) emulsion as described by^44^. However, some formulations were developed by solid in oil in water S/O/W. Briefly, the w_1_ phase consisting of protein and trehalose dissolved in ultra-pure water (>Ω18) was dispersed into the ethyl acetate containing 1.67% w/v copolymer and 6% Span^60^ by probe sonication. Then, W/O emulsion was added to 50 ml of 3% PVA/water to prepare the secondary emulsion by probe sonication. Afterwards, the double emulsion was stirred overnight at the room temperature to evaporate the organic solvent and solidify the nanocapsules. The residues of organic solvent and free proteins were removed by centrifuging and washing the nanocapsules for times for 30 minutes at 15000 rpm and 4 °C before the lyophilization for 48 hours.

For the S/O/W preparations, the protein and trehalose were directly suspended in the organic phase and sonicated to prepare the finely dispersed S/O suspension. Then, all the remaining steps same as W/O/W preparation method.

### Encapsulation efficiency of proteins

Encapsulation efficiencies of the different formulations were measured by applying the previously developed method^45^. The polymeric shells were broken by suspending and stirring the nanocapsules in ethyl acetate under fume cupboard for 2 hours. Then, it was centrifuged 2 times for 10 minutes at 10000 rpm; the pellets were collected and left under the fume cupboard for half an hour to dry and then suspended in water and stirred for 2 hours until all the protein is dissolved. The suspension was centrifuged at 10000 rpm for 10 min, and then the supernatant was analysed, by using SEC, to determine the protein concentration in the sample.

### The particle size of protein polymeric nanocapsules measurement

Zeta PALS^®^ dynamic light scattering was utilised to determine the nanocapsules containing proteins particle size. Samples were prepared by suspending 10 mg of the freeze-dried nanocapsules in 5 ml water. Afterwards, the resulting suspensions were mixed in the vortex for 1 minute and then were left in water bath sonicator for 5 minutes. Nanocapsule diameters were measured in triplicate at 25 °C.

### Microscopic imaging of polymeric nanocapsules using Negative Staining Transmission Electron Microscopy (TEM)

The morphology of PNCs was investigated by TEM. The technique was applied by using negative staining technique 1% (w/v) of sodium silicotungstate solution. A drop of PNCs suspension was applied on a 400 mesh Formvar copper grid on paraffin, and the sample was adhered on the Formvar at room temperature for 15 minutes, then, a drop of 1% (w/v) of sodium silicotungstate solution was applied for 5 minutes. The obtained specimen was later observed under the TEM.

### *In vitro protein* release in Simulated Gastric Fluid (SGF) and Simulated Intestinal Fluid (SIF) without enzymes

Protein release from the nanocapsules was determined in simulation gastric fluids (SGF) and simulated intestinal fluid (SIF) for 4 and 24 hours, respectively. Both SIF and SGF were prepared as per British Pharmacopeia 2014. However, no digestive enzymes were added.

Powder nanocapsules containing 1.5 mg entrapped protein, calculated based on relevant encapsulation efficiency, were suspended in 30 ml SIF (pH 6.8), and in SGF (pH 1.2), then this volume was divided into 10 screw cap Eppendorfs, each contains 2 ml (100 μg protein). The vials were incubated in a shaker water bath under shaking rate of 50 cycles/minute at 37 °C, and one vial was collected at each time point. All release tests were performed in triplicate.

Eppendorf vials were taken, and the samples centrifuged 2 times at 10000 rpm for 10 minutes, the pellets were collected, and the shells were broken down. Then the remaining unreleased proteins were collected. Protein release was determined by quantification of protein amount remaining in pellets by using SEC after pellets disruption and subtracting the remaining from the original value (100 μg) to find out release percentage.

### Measurement of biological activity of the encapsulated protein

The effect of the used reagents including polymers and encapsulation process on protein biological activities was examined in this study. PNCs shell was disrupted by adding ethyl acetate, and the biological activity of the encapsulated lysozyme was determined by its ability to lysis the bacterial cell wall. The calculation was performed by recording ΔA450 for the bacterial-lysozyme suspension over 5 minutes, as described previously by^46^. The biological activity of DNase I in this study was detected by applying the enzymatic assay procedures established by *Kunitz 1950*^47^. The rate of the cleaving of phosphodiester linkage of DNA is considered as a function of DNAse I activity^48^.

## Electronic supplementary material

Supplementary Information
