# Supplementary material for: Lysozyme and DNase I loaded poly (D, L lactide-co-caprolactone) nanocapsules as an oral delivery system
Source: Sci Rep. 2018 Sep 3;8:13158. doi: 10.1038/s41598-018-31303-x (PMC6120872; doi:10.1038/s41598-018-31303-x)
Supplement: Supplementary file 1 — Supplementary Information [file 41598_2018_31303_MOESM1_ESM.docx]

Supplement Information

**Title: Lysozyme and DNase I loaded poly (D, L lactide-co-caprolactone) nanocapsules as an oral delivery system.**

**Authors:** Omar S. Abu Abed*, Cheng Chaw, Lee Williams, Amal A. Elkordy

**Affiliations:** Department of Pharmacy Health & Well-being, Faculty of Health Sciences and Wellbeing, University of Sunderland, Sunderland, SR1 3SD, UK.

*Corresponding author, [Omar.abuabed@gmail.com](mailto:Omar.abuabed@gmail.com)

**Supplementary Information Figure Legends**

**Figure S1. Particle size distribution of PNCs.**

**Figure S2. Lysozyme calibration curve.**

**Figure S3. FTIR Spectra of lysozyme dissolved in SGF and SIF.**

**Figure S4. HPLC chromatogram for detection of trehalose encapsulation.**

**Supplementary Information Table Legends**

**Table S1.** The coefficients of the factors affecting the PNCs characteristics, their interaction along with P values for each factor.

| **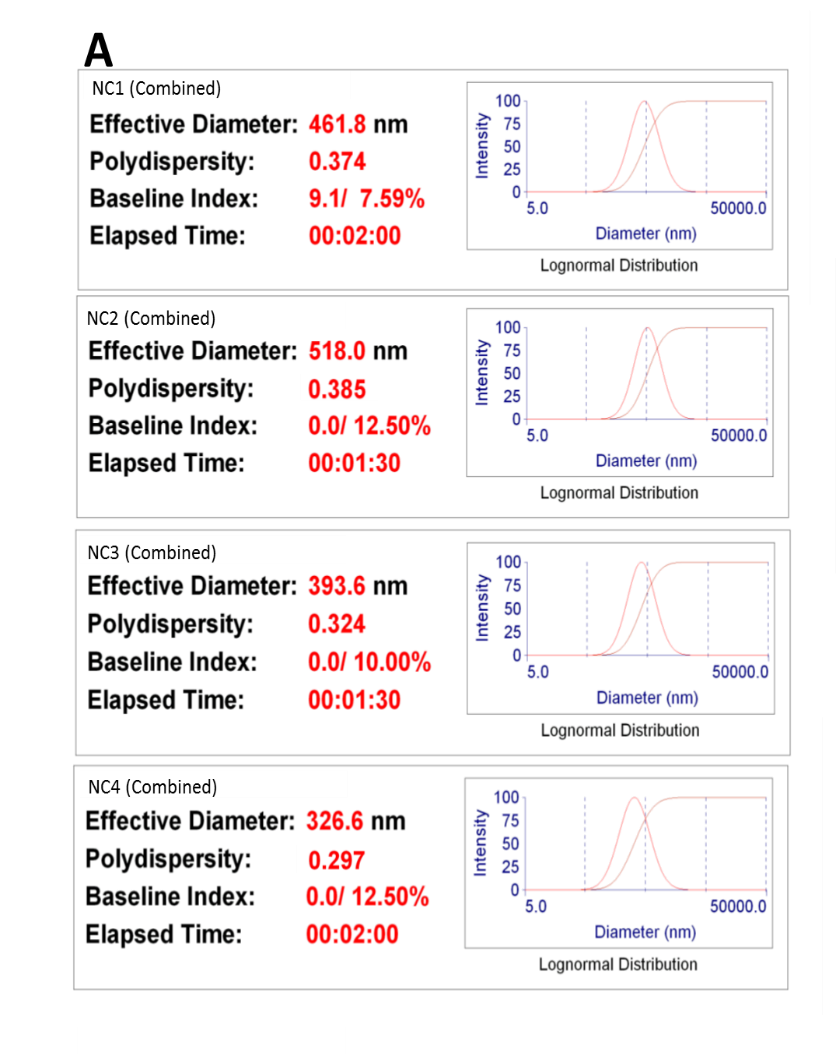** | **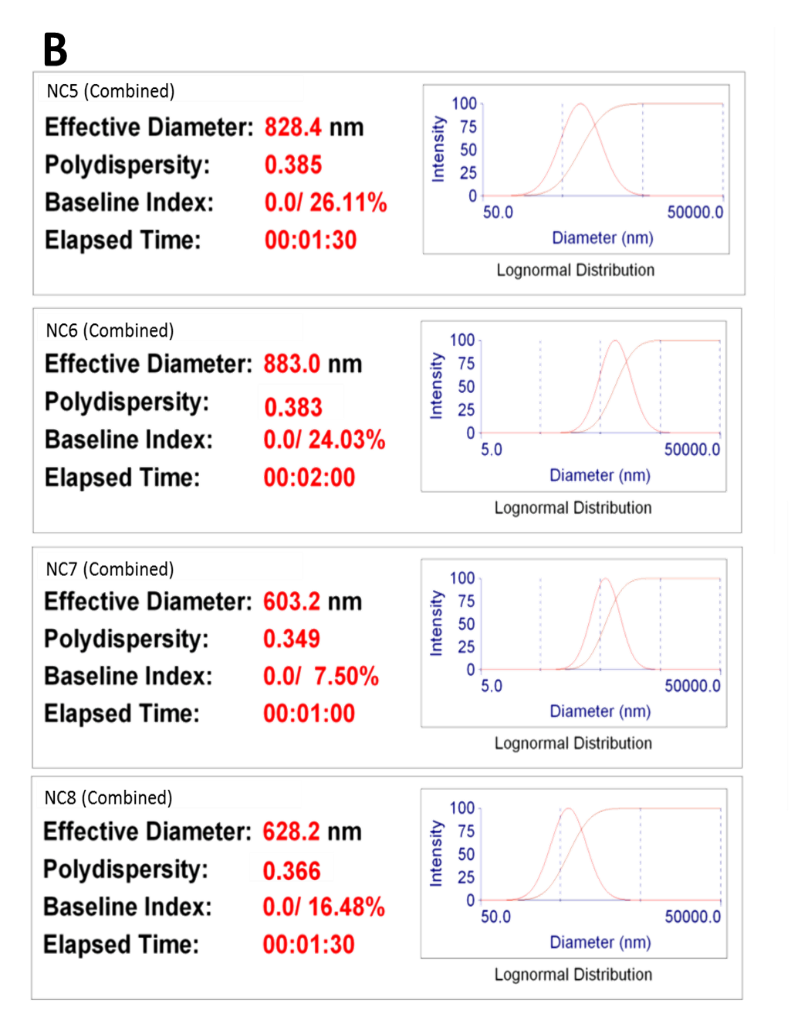** |
| --- | --- |

**Figure S1. Particle size distribution of PNCs.** The particle size of the prepared PNCs was examined by utilizing dynamic light scattering zeta sizer. The investigation of particle size was performed in water (viscosity 0.890 cP) at 25 °C with 1.330 Ref. Index Fluid, 90-degree angle, and 660.0 nm wavelength. Three runs were completed with an average time of 30 seconds for each run, and the average count rate was 79.0 kcps. a. Particle size distribution of four PNC formulations prepared without trehalose. b. Particle size distribution of four PNC prepared with trehalose.

**Figure S2. Lysozyme calibration curve.** The standard curve was generated by plotting a serial concentration (10 µg/ml- 200 µg/ml) vs. area under the curve for each relevant concentration obtained from HPLC. Three replicate injections of each concentration were analysed, averages and standard deviation were calculated. The linear regression and correlation coefficient were calculated from the graph between peak area and concentration. The analysis was carried out on an integrated Agilent 1100 HPLC with an infinity UV-diode array detector (DAD) at 214 nm (Agilent Technologies, Delaware, USA), using a size exclusion column (Agilent SEC-5,100A, 7.8x150mm) with internal temperature 25 °C. Isocratic separation system was used with a mobile phase constituent of 150 mM Sodium phosphate buffer pH 7 at a flow rate 1 ml/minute and 10 µl injection volume for a total chromatographic run time 10 minutes. Peak areas and retention times were obtained by utilising Agilent Chemstation® software.

**
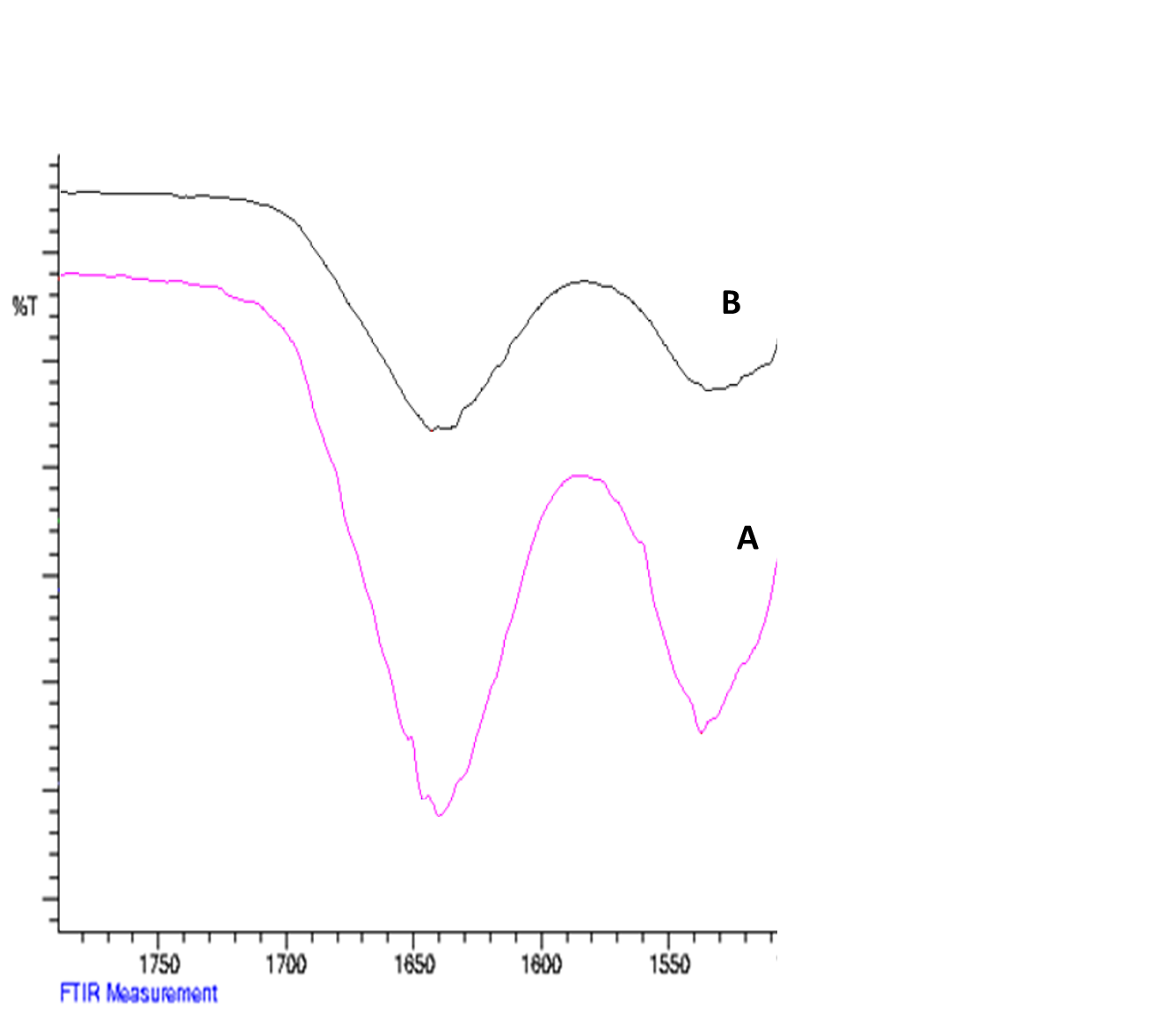
**

**Figure S3. FTIR Spectra of lysozyme dissolved in SGF and SIF.** FTIR spectroscopy was used to investigate the secondary structure of lysozyme after the exposure to SGF and SIF to investigate whether the extremely acidic medium (SGF) has affected the secondary structure of lysozyme or not. FTIR spectroscopy was performed using IR Affinity-1S (SHIMADZU, UK). The FTIR spectra were obtained after subtraction of the background from 4000 to 550 cm-1 at 4 cm-1 resolution and interval of 2 cm ^-1^. Each measurement was an average of 50 scans at room temperature (22 ± 2 ˚C). A drop of each sample solution was directly loaded into the system and analysed. Peak labelling and positions of the spectra were analysed using the spectrum software (SHIMADZU, Inc, UK). a. FTIR spectrum of lysozyme released in SIF at pH 6.8; Amide I and Amide II peaks were found at 1645.28 and 1548.63, respectively. b. FTIR spectrum of lysozyme released in SGF at pH 1.2; Amide I and Amide II peaks were found at 1647.21 and 1550.41, respectively. No significant shift of Amide I or Amide II was noticed due to pH changes.

**
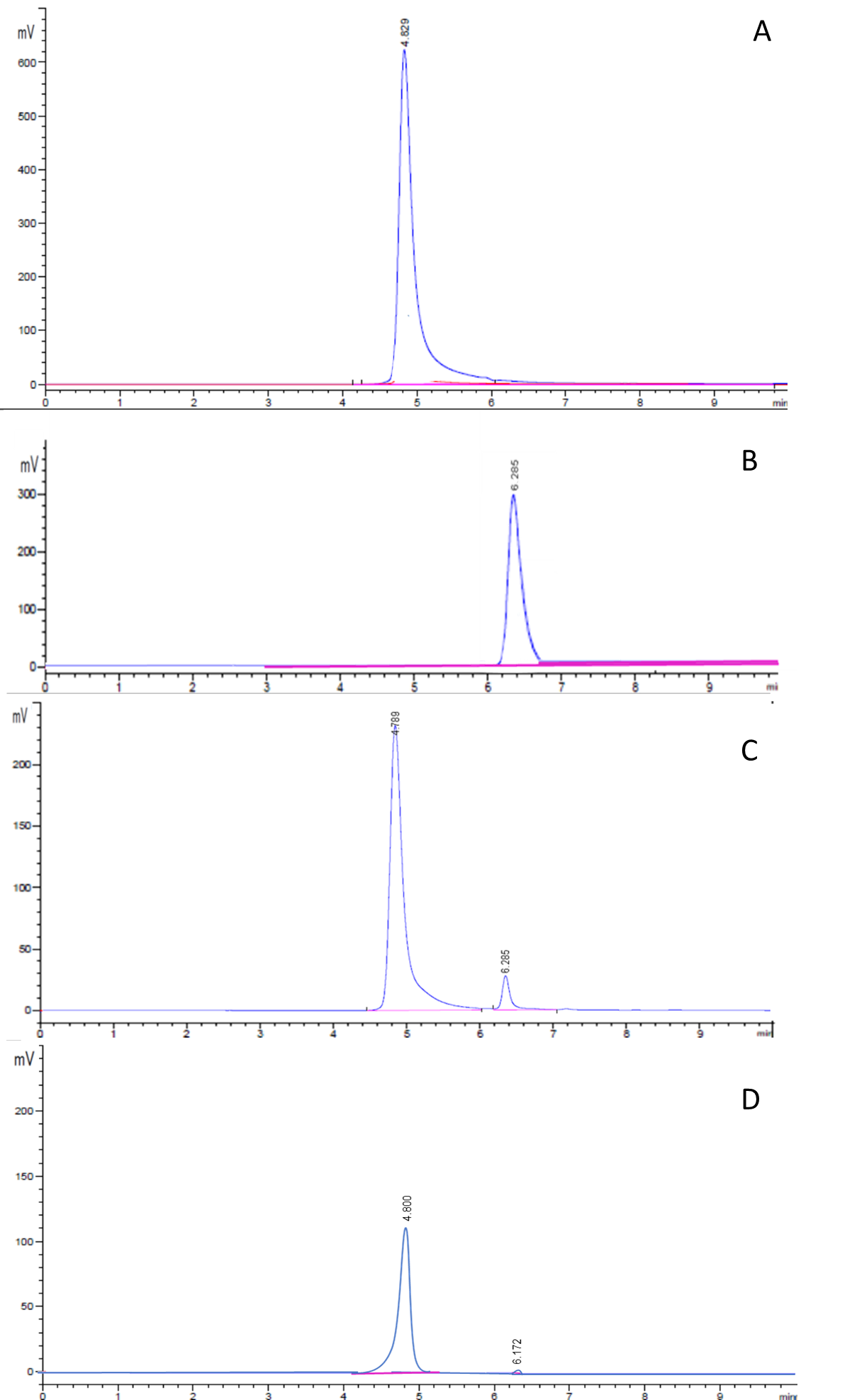
**

**Figure S4: HPLC chromatogram for detection of trehalose encapsulation.** HPLC-SEC chromatography was employed to detect the presence of trehalose in the aliquot obtained after shell breaking and collection of encapsulated materials. The analysis was carried out on an integrated Agilent 1100 HPLC with an infinity 1260 Evaporative Light Scattering Detector (Agilent Incorporation, Delaware, USA) at 60 °C evaporative temperature, gas flow rate 2 SLM (standard litre per minutes) and PMT gain 1/40 Hz, using a size exclusion column (Agilent SEC-5,100A, 7.8x150mm) with internal temperature of 25 °C. Isocratic separation system was used with a mobile phase constituent of 150 mM Sodium phosphate buffer pH 7 at a flow rate 1 ml/minute and 10 µl injection volume for a total chromatographic run time 10 minutes. Peak areas and retention times were obtained by utilising Agilent Chemstation® software. a. Chromatogram of pure lysozyme prepared in water (1mg/ml). b. Chromatogram of pure trehalose prepared in water (10 mM). c. Chromatogram of encapsulated lysozyme and trehalose for PNC7 which contains 10mM. d. Chromatogram of encapsulated lysozyme and trehalose for PNC3 which contains 1mM. The chromatograms confirm the encapsulation of trehalose in the core of PNC as the peaks for trehalose appear at retention time around 6.20 minutes.

**Table ‎S1.** The coefficients of the factors affecting the PNCs characteristics, their interaction along with P values for each factor.

|  | **EE** | | **Release in SIF** | | **Particle Size** | | **Biological activity** | |
| --- | --- | --- | --- | --- | --- | --- | --- | --- |
| **Factor** | Coefficient | P | Coefficient | P | Coefficient | P | Coefficient | P |
| Polymer ratio | 14.975 | 0.011 | -17.223 | 0.047 | 7.149 | 0.659 | -0.205 | 0.631 |
| Trehalose | 0.500 | 0.295 | 0.802 | 0.643 | 154.6 | 0.049 | 18.846 | 0.011 |
| Core physical state (Solid) | 0.775 | 0.198 | -0.227 | 0.881 | -93.775 | 0.081 | 8.602 | 0.023 |
| Polymer ratio * Trehalose | 0.150 | 0.656 | 1.667 | 0.416 | 7.725 | 0.638 | 0.153 | 0.710 |
| Polymer ratio * Core physical state (Solid) | -3.575 | 0.044 | 0.908 | 0.607 | -13.900 | 0.455 | 1.261 | 0.155 |
| Trehalose * Core physical state (Solid) | 0.500 | 0.295 | 1.676 | 0.415 | -23.450 | 0.303 | 0.544 | 0.333 |

P= P-value, p≤0.5.

Red colour for significant factors.

*Interaction between factors.
